# Subclinical myocardial dysfunction in pituitary neuroendocrine tumor patients: a 2D speckle-tracking echocardiography study

**DOI:** 10.1530/EC-25-0641

**Published:** 2025-12-09

**Authors:** Yinxia Li, Yifei Yu, Yanxin Xu, Rui Shen

**Affiliations:** ^1^ Department of Ultrasound, Shanghai Municipal Hospital of Traditional Chinese Medicine, Shanghai University of Traditional Chinese Medicine, Shanghai, China; ^2^ Department of Endocrinology, Huashan Hospital Affiliated to Fudan University, Shanghai, China; ^3^ Emergency Pharmacy Department, Huashan Hospital Affiliated to Fudan University, Shanghai, China

**Keywords:** echocardiography, 2D-STE, PitNETs, left ventricular global longitudinal strain, myocardial injury

## Abstract

Pituitary neuroendocrine tumors (PitNETs) cause hormonal hypersecretion, which can disrupt cardiovascular homeostasis and lead to both overt and subclinical cardiac dysfunction. This study retrospectively examined 198 hospitalized PitNET patients (*n* = 91 male, *n* = 107 female) from March 2019 to December 2022. Two-dimensional speckle-tracking echocardiography (2D-STE) was used to determine the absolute left ventricular global longitudinal strain (GLS) values. Comprehensive clinical information was gathered, including endocrine axis status, symptoms, surgery history, and demographics. Standard transthoracic echocardiography parameters were also recorded. Multiple linear regression analysis and intergroup comparisons were used to identify factors affecting GLS. Pre-planned subgroup analyses were conducted for the acromegaly cohort and by surgical status. Clinical presentations overlapped across tumor subtypes. Cranial imaging was the primary method for detecting cases with atypical symptoms. Female patients were older and sought treatment more frequently than males. The mean patient age was 43.7 years, with most diagnoses occurring between 30 and 50 years. GLS showed a significant positive correlation with EF (62.64 ± 5.04%; *P* = 0.00) and E/e' ratio (7.10 ± 1.96; *P* = 0.01), and a significant negative correlation with BMI (25.20 ± 3.92 kg/m^2^; *P* = 0.00). Significant differences in GLS were observed based on gender (*t* = 2.47, *P* = 0.01) and operator (*t* = 2.52, *P* = 0.01). Regression analysis identified baseline predictors (BMI), EF, LV function, and gender as independent predictors of GLS. A key finding from a dedicated acromegaly subgroup analysis was that disease activity (IGF-1) independently predicted impaired GLS. 2D-STE is a sensitive tool for detecting subclinical myocardial dysfunction in PitNET patients. GLS is significantly influenced by BMI, EF, left ventricular dimensions, and gender, underscoring the need to integrate metabolic and cardiac profiles into the cardiovascular assessment of this population. Furthermore, the identification of IGF-1 as a predictor in acromegaly highlights the value of disease-specific cardiac risk stratification.

## Introduction

Pituitary neuroendocrine tumors (PitNETs), formerly known as pituitary adenomas, are the second most common intracranial tumor and arise from the master endocrine pituitary gland. While typically benign, nearly 40% exhibit invasive behavior, and a refractory subset resists conventional treatment; the rare (0.2%) metastatic forms are classified as pituitary carcinomas (1). With a population prevalence of approximately 17%, these tumors often elude timely diagnosis, with delays of 5–25 years common due to their insidious symptomatic onset (2, 3). The contemporary WHO classification categorizes PitNETs into functional and non-functional types based on cell lineage, pituitary-specific positive transcription factor 1 (PIT1), T-box transcription factor (TPIT), or steroidogenic factor 1 (SF1), with variants including immunonegative and plurihormonal tumors. This diversity in hormonal secretion creates a complex clinical landscape, complicating diagnosis and frequently leading to significant treatment delays (4, 5).

The anterior pituitary is central to neuroendocrine function, primarily mediated through the hypothalamic-pituitary-adrenal (HPA), hypothalamic-pituitary-gonadal (HPG), and hypothalamic-pituitary-thyroid (HPT) axes. The hypothalamus produces corticotropin-releasing hormone, gonadotropin-releasing hormone (GnRH), and thyrotropin-releasing hormone. These act on the anterior pituitary to stimulate the release of adrenocorticotropic hormone (ACTH), gonadotropins (luteinizing hormone and follicle-stimulating hormone), and thyroid-stimulating hormone (TSH), which in turn regulate the synthesis and secretion of glucocorticoids (e.g., cortisol), gonadal steroids (e.g., estradiol, progesterone, testosterone), and thyroid hormones (T3, T4) (6).

Beyond these axes, hypothalamic growth hormone-releasing hormone (GHRH) stimulates the anterior pituitary to synthesize and release growth hormone (GH) (7). GH regulates normal cell growth and metabolism. Both estrogens and androgens stimulate its secretion; however, estrogens inhibit its hepatic effects while androgens enhance them, promoting the production of insulin-like growth factor-1 (IGF-1) (8). As an anabolic hormone, IGF-1 decreases with age, a trend associated with increased inflammation and elevated fibrinogen, suggesting low levels may elevate cardiovascular risk (9, 10). Regular GH and IGF-1 secretion helps maintain vascular and cardiovascular health. In contrast, GH excess leads to gigantism or acromegaly, increasing the risk of diabetes, hypertension, and cardiac hypertrophy (11). Similarly, cortisol excess from corticotropin-producing tumors (Cushing’s syndrome) causes metabolic abnormalities and significantly elevates the risk of atherosclerosis, coronary artery disease, and heart failure, resulting in lasting cardiac changes (12). Finally, thyrotropin-producing tumors cause excessive thyroid hormone production, which disrupts cardiovascular hemodynamics and can lead to high-output heart failure or dilated cardiomyopathy (13).

Given the established cardiovascular sequelae of hormonal excess, sensitive tools for early detection are critical. Two-dimensional speckle-tracking echocardiography (2D-STE) is a novel technique for the quantitative assessment of myocardial function. It analyzes the motion of acoustic speckles on conventional two-dimensional images to evaluate myocardial deformation accurately. A key advantage of 2D-STE over traditional echocardiography is its ability to assess global and regional myocardial strain quantitatively. This allows for detecting subclinical cardiac impairment not identifiable with conventional methods, thereby providing valuable diagnostic and prognostic information for early disease diagnosis (14, 15).

Therefore, given that functional PitNETs can induce cardiac structure and function alterations, the systematic approach to diagnosis, treatment, and follow-up within our hospital’s integrated endocrinology and neurosurgery department provides a unique opportunity for investigation. This study collected case data from newly diagnosed and post-operative pituitary tumor patients. We utilized 2D-STE to measure left ventricular global longitudinal strain (GLS) and assess subclinical myocardial changes. Our primary aim was to elucidate the relationship between these subclinical changes, particularly in functional PitNETs, and the patients’ specific clinical characteristics and pathophysiological states. Ultimately, this research seeks to establish the utility of left ventricular GLS as an echocardiographic biomarker for the early diagnosis and intervention of pituitary tumor-related cardiac impairment.

## Methods and techniques

### Study population

A total of 198 patients admitted to the integrated pituitary tumor ward of our hospital’s endocrinology and neurosurgery department between March 2019 and December 2022 were enrolled in this study. The cohort included 91 males and 107 females, with a mean age of 43.74 ± 13.21 years. The cohort comprised a mixed population of PitNETs, including acromegaly (GH-secreting; *n* = 164), Cushing’s disease (ACTH-secreting; *n* = 7), and non-functioning pituitary tumors (NFPTs; *n* = 16). This study was conducted per the Declaration of Helsinki and was approved by the Ethics Committee of Huashan Hospital, Fudan University (approval no: 2018002), and a written informed consent was obtained from all participants.

Inclusion criteria required a confirmed diagnosis of pituitary tumor and presentation for either initial diagnosis or routine post-operative follow-up. The specific conditions of evaluation were defined as follows: ‘initial diagnosis’ referred to treatment-naïve patients, while ‘routine post-operative follow-up’ referred to patients studied during a stable clinical state more than 3 months after transsphenoidal surgery. For post-operative patients, the time since surgery was recorded and categorized as ≤6 months or >6 months to evaluate potential long-term effects of tumor resection on myocardial function. These intervals were used in the statistical analyses to assess differences in GLS between pre-operative and post-operative patients, as well as to investigate time-dependent changes in post-operative cardiac remodeling.

Exclusion criteria encompassed a history of coronary artery stent implantation, congenital heart disease, acquired valvular diseases (e.g., mitral valve prolapse), or reduced cardiac function, defined as an ejection fraction (EF) below 52% for males and 54% for females, or a diagnosis of heart failure. Additional exclusions were segmental wall motion abnormalities, myocardial disease unrelated to pituitary tumors, autoimmune diseases with cardiac, hepatic, or renal involvement, atrial fibrillation or other arrhythmias, pacemaker implantation, and insufficient echocardiographic image quality. General data and relevant hematologic test results of the pituitary tumors were recorded. Upon admission, every participant provided written informed consent for the research.

### Echocardiographic assessment

All patients were connected to a standard three-lead electrocardiogram before the echocardiographic examination, which commenced once the waveform stabilized. A complete examination was performed using a PHILIPS EPIQ-7C ultrasound system with a 3–7 MHz phased-array transducer.

Two experienced echocardiographers, randomly assigned to each patient, performed all transthoracic echocardiography (TTE) and 2D-STE assessments. After reviewing the clinical request form and inquiring about the patient’s cardiac history, the examiner positioned the patient in the left lateral decubitus position. Using M-mode ultrasound in the parasternal long-axis view, the left ventricular end-diastolic diameter, end-systolic diameter, left ventricular ejection fraction (LVEF), left atrial diameter, and the diastolic thickness of the interventricular septum were measured. If the long-axis view was suboptimal, LVEF was calculated using the modified Simpson’s method from the apical four-chamber (A4C) and two-chamber (A2C) views.

From the A4C view, pulse-wave Doppler was used to acquire the mitral valve inflow early (E) to late (A) diastolic peak velocity ratio (E/A) and the ratio of early mitral inflow velocity to the average early diastolic tissue Doppler velocity (e′) of the mitral annulus and interventricular septum (E/e′). Color Doppler imaging was applied across all standard views to assess mitral, tricuspid, and aortic valve regurgitation. Higher gain and lower depth settings were employed to ensure clear endocardial visualization. Patients were instructed to hold their breath briefly during image acquisition to minimize motion artifact.

**Figure 1:**
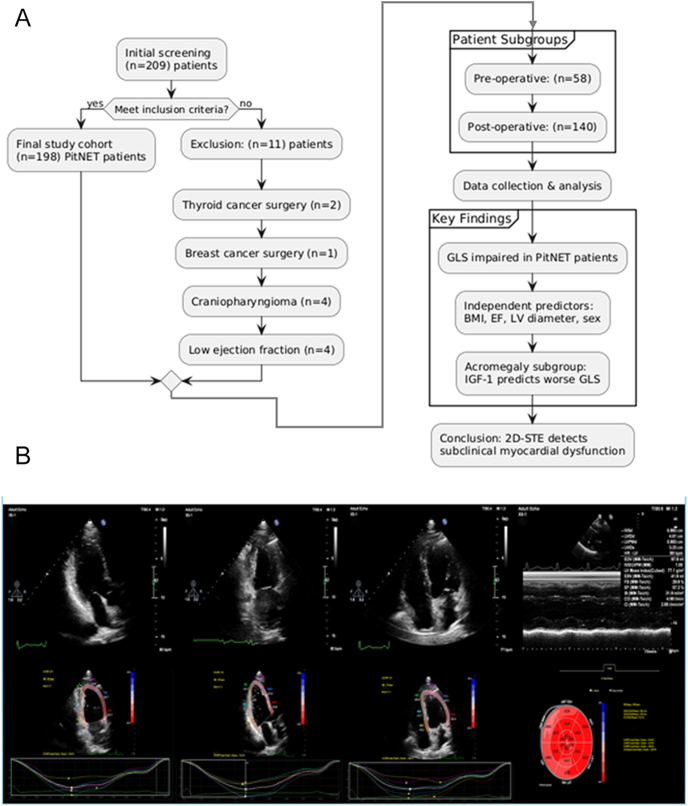
Workflow, image analysis, and bull’s-eye display of 2D-STE.

Once satisfactory images of the standard apical four-chamber (A4C), two-chamber (A2C), and three-chamber (A3C) views were acquired, the machine’s integrated cardiac motion quantification plugin (auto cardiac motion quantification, aCMQ) was activated for 2D-STE analysis in accordance with established methodologies (16). The QLab software package automatically tracks three points on the mitral annulus and the opposite septal mitral annulus for strain analysis. The region of interest (ROI) was manually adjusted for segments with poor tracking. The left ventricular GLS was calculated as the average value over three consecutive cardiac cycles. The left ventricle was divided into 17 segments, and the GLS values from the A3C, A4C, and A2C views were used to generate a bull’s-eye plot. Patients with unsatisfactory image quality in any apical view or with two or more segments exhibiting poor tracking were excluded from the analysis. Representative images of the acquisition, the resulting bull’s-eye plot, and a study flowchart are provided in Fig. 1.

### Statistical analysis

All statistical analyses were performed using IBM SPSS Statistics (version 23.0) and the R software environment (version 4.4.0; https://www.R-project.org). The ggplot2 package in R was utilized to generate fitted linear and box plots for data visualization. Continuous variables are presented as mean ± standard deviation, and categorical variables are expressed as numbers and percentages. Differences between two groups of continuous variables were assessed using an independent samples *t*-test. A one-way analysis of variance (ANOVA) was applied for comparisons across more than two groups. A *P*-value of less than 0.05 was considered statistically significant for all group comparisons. The linear association between continuous variables was evaluated using Spearman’s rank correlation coefficient.

#### Regression modeling

To identify independent predictors of left ventricular GLS, generalized linear regression models were constructed. Variables with *P* < 0.1 in univariate analyses were considered for inclusion in the initial multivariable model. Model assumptions, including linearity, homoscedasticity, normality of residuals, and absence of multicollinearity (all variance inflation factors <10), were verified.

#### Pre-planned subgroup analyses

To address cohort heterogeneity and potential surgical bias, the following pre-planned subgroup analyses were performed:

Acromegaly-specific analysis: a dedicated multivariable linear regression model was constructed for the GH-secreting (acromegaly) subgroup (*n* = 164) to identify tumor-specific predictors. The model included BMI, EF, left ventricular end-diastolic diameter (LVEDD), IGF-1 levels, sex, and operator.

Surgical status analysis: patients were stratified into pre-operative (treatment-naïve) and post-operative groups. Post-operative patients were further subdivided by follow-up interval (≤6 months vs >6 months). GLS values were compared between these strata using *t*-tests and ANOVA.

## Results

### Population demographics and tumor classification

A total of 209 patients initially underwent a valid 2D-STE assessment for left ventricular GLS. After excluding 11 patients based on predefined criteria, including a history of thyroid malignancy surgery (*n* = 2), breast cancer surgery (*n* = 1), craniopharyngioma (*n* = 4), or subnormal ejection fraction (*n* = 4), 198 patients were included in the final analysis. The cohort was composed of the following tumor subtypes: GH-secreting (acromegaly; *n* = 164, 82.8%), ACTH-secreting (Cushing’s disease; *n* = 7, 3.5%), TSH-secreting (*n* = 4, 2.0%), gonadotropin-secreting (*n* = 3, 1.5%), mixed-secreting (*n* = 4, 2.0%), and non-functional PitNETs (*n* = 16, 8.1%).

Symptom profiles differed based on clinical status: treatment-naïve patients typically presented with signs of endocrine hypersecretion (e.g., acral or facial changes, Cushingoid appearance, or hyperthyroid symptoms), whereas post-operative patients, assessed during stable follow-up, most commonly presented with findings related to hypopituitarism or partial symptom remission. All diagnoses were confirmed via endocrinology and neurosurgery referrals, with head imaging (e.g., MRI) as the principal detection method; detailed clinical presentations for each tumor subtype are provided in Table 1.

**Table 1:** Frequency and clinical characteristics of the study cohort by PitNET type.

PitNET type	Number of cases	Total cohort (%)	Patient complaints at presentation
Functional PitNETs			
GH-secreting (acromegaly)	164	82.8	Acral enlargement, facial coarsening, excessive sweating, joint pain, headache, visual impairment, amenorrhea, and galactorrhea
ACTH-secreting (Cushing’s)	7	3.5	Weight gain, easy bruising, muscle weakness, emotional changes, polyuria/polydipsia
TSH-secreting	4	2.0	Palpitations, tremors, heat intolerance, irritability, and weight loss
Gonadotropin-secreting	3	1.5	Visual impairment, headache, and menstrual irregularities
Mixed secretion	4	2.0	Combination of symptoms from relevant hormonal excess syndromes (e.g., acromegaly and hyperthyroidism)
Non-functional PitNETs			
Non-functional pituitary tumor	16	8.1	Visual impairment, headache, dizziness, fatigue, loss of libido
**Total**	**198**	**100.0**	

### Age distribution

**Figure 2:**
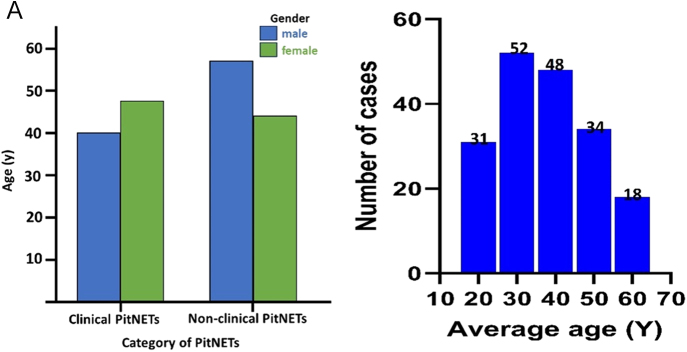
Age distribution in functional versus non-functional PitNETs.

The study population comprised 91 male patients (mean age 41.96 ± 12.53 years) and 107 female patients (mean age 45.25 ± 13.63 years). Notably, the female patients were both more numerous and older on average than the male patients. Next, we compared the age distribution between functional and non-functional PitNETs; the distribution for functional PitNETs is shown in Fig. 2. The age at diagnosis was most frequent between 30 and 50 years.

### GLS values in functional and non-functional PitNET patients

The average GLS value in the 198 patients included in the study (comprising both functional and non-functional PitNETs) was 14.26% ± 2.83, significantly lower than the usual lower limits of −16.9% for adult males and −18.5% for adult females. Among continuous variables, GLS showed a significant positive correlation with left ventricular ejection fraction (LVEF; 62.64% ± 5.04, *P* = 0.00) and the E/e' ratio (7.10 ± 1.96, *P* = 0.01). No statistically significant correlation was observed between GLS and left atrial diameter or interventricular septal thickness; a positive correlation with LVEDD was noted but was not statistically significant (*P* > 0.05). Among general data, body mass index (BMI; 25.20 ± 3.92) showed a significant negative correlation with GLS (*P* = 0.00), whereas other variables, including insulin-like IGF-1 levels, showed no significant correlation (*P* > 0.05) (Table 2).

**Table 2:** Correlation analysis between GLS and continuous variables.

Variable	Mean ± SD	*r*-value	*P*-value
GLS	14.26 ± 2.83	−0.02	0.76
Age	43.74 ± 13.21	−0.20	0.00*
BMI	25.20 ± 3.92	0.27	0.00*
EF	62.64 ± 5.04	0.13	0.06
E/A	1.05 ± 0.30	0.17	0.02*
E/e	7.10 ± 1.96	0.04	0.59
LA	35.64 ± 4.16	0.14	0.06
LVDD	48.19 ± 4.94	−0.04	0.56
IVS	10.02 ± 1.27	−0.02	0.75
IGF-1	383.24 ± 253.81	−0.02	0.76

GLS, global longitudinal strain; BMI, body mass index; EF, ejection fraction; LA, left atrial diameter; LVDD, left ventricular end-diastolic diameter; IVS, interventricular septal thickness; IGF-1, insulin-like growth factor 1.

* Statistical significance at *P* < 0.05.

### Association between clinical and echocardiographic characteristics and GLS in PitNET patients

The clinical data of the patients were categorized based on various factors. These included tumor functionality (functional or non-functional PitNETs), surgical treatment status, post-surgical outcomes (biochemical remission or non-remission), and pre- and post-surgical symptoms. Symptoms were classified as none, pituitary dysfunction, abnormal glucose tolerance, and/or abnormalities in blood pressure or lipids, with or without pituitary dysfunction. Additional factors included gender, operator (C or L), time from onset to first diagnosis (less than 3 years/over 3 years), the functional status of the HPG, HPA, and HPT axes (normal or reduced), prolactin levels (normal/increased/decreased), time of first diagnosis or post-surgical follow-up (≤6 months/>6 months), and the presence of mild or greater valve regurgitation. Intergroup comparisons revealed significant differences in the absolute GLS values based on gender (*t* = 2.47, *P* = 0.01) and operator (*t* = 2.52, *P* = 0.01). However, there were no statistically significant differences for surgical treatment, biochemical remission, prolactin levels, symptoms, or follow-up time (*P* > 0.05) (Table 3; Supplementary Fig. S1 (see section on Supplementary materials given at the end of the article)).

**Table 3:** Comparison of GLS by gender and operator.

Variable	Group	Cases	Absolute value of GLS	*t*/*F* value	*P*
Sex	Male	91	13.72 ± 2.85		
	Female	107	14.71 ± 2.75	2.47	0.01*
Category	Clinical PitNETs	182	14.25 ± 2.85		
	Nonclinical PitNETs	16	14.31 ± 2.65	−0.08	0.94
Surgery	Operation	132	14.15 ± 2.81		
	Nonoperation	66	14.47 ± 2.89	−0.74	0.46
Handler	C	84	14.84 ± 2.86		
	L	114	13.83 ± 2.75	2.52	0.01*
Interval	≤3Y	92	13.90 ± 2.78		
	>3Y	106	14.57 ± 2.85	−1.66	0.09
HPA-axis	Normal	116	14.24 ± 2.80		
	Lower	82	14.28 ± 2.89	−0.12	0.91
HPT-axis	Normal	145	14.29 ± 2.80		
	Lower	53	14.15 ± 2.95	−0.32	0.75
HPG-axis	Normal	139	14.37 ± 2.87		
	Lower	59	13.98 ± 2.74	0.88	0.38
Regurgitation	NO	168	14.22 ± 2.80		
	Yes	30	14.44 ± 3.04	−0.39	0.70
PRL	Upper	78	14.35 ± 2.83		
	Normal	106	14.22 ± 2.86		
	Lower	14	14.01 ± 2.86	0.10	0.90
Effect	Nonoperation	58	14.59 ± 2.85		
	Remission	77	14.40 ± 2.78		
	No remission	63	13.76 ± 2.85	1.48	0.23
Complication	Nothing	62	14.74 ± 3.25		
	Hypopituitarism	42	13.95 ± 2.87		
	Disturbance	70	13.99 ± 2.63		
	Both	24	14.32 ± 2.04	0.96	0.41
Time	Pre-operation	58	14.59 ± 2.85		
	Post-operation ≤6M	61	14.54 ± 2.85		
	Post-operation >6M	79	13.78 ± 2.77	1.85	0.16

HPA, hypothalamic-pituitary-adrenal; HPT, hypothalamic-pituitary-thyroid; HPG, hypothalamic-pituitary-gonadal; PRL, prolactin.

* Statistical significance at *P* < 0.05.

### Inter-operator variability in measurements

**Figure 3:**
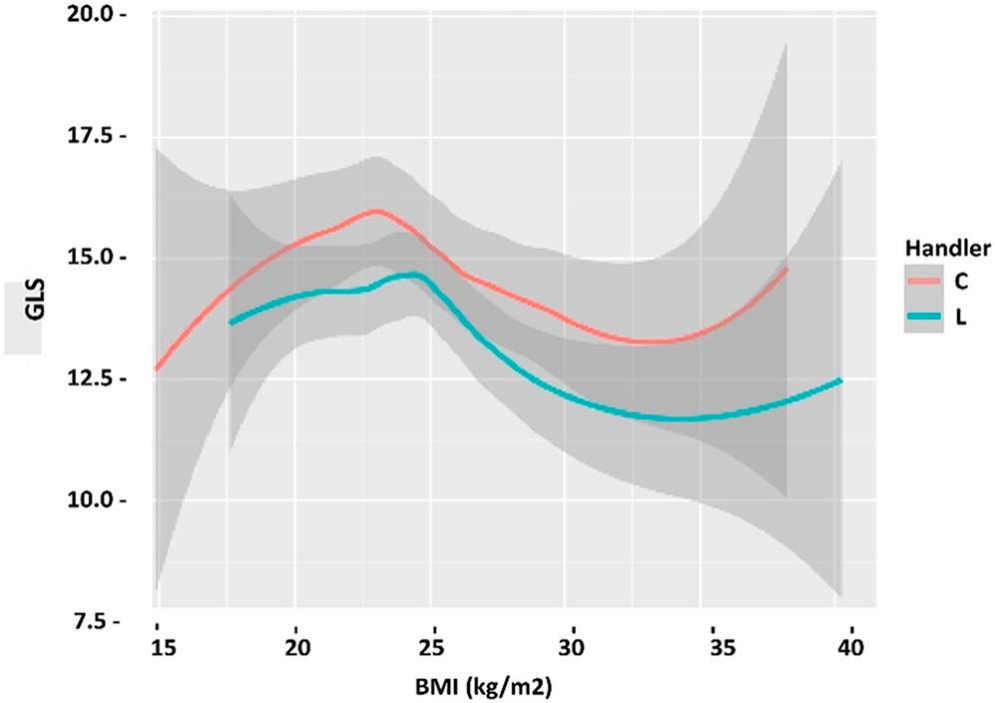
Linear regression analysis of GLS measurements by different operators.

The differences between operators were assessed using linear mapping. A linear regression plot was created for the absolute BMI, EF, E/e, and GLS values, corresponding to different operators. The plot revealed that the average GLS value measured by operator C was higher than that estimated by operator L. This discrepancy was also observed in both operators’ BMI, EF, and E/e values (Fig. 3).

### Multiple linear regression analysis

A multiple linear regression analysis was conducted using variables with a *P*-value of less than 0.1. The study revealed a linear association between the GLS absolute values and BMI, EF, E/A, E/e, LVEDD, sex, operator (C, L), and the interval between the onset of the disease and the first visit (interval), as indicated by partial regression scatter plots and scatter plots of studentized residuals versus predicted values. These variables were also confirmed to be independent (Durbin-Watson test value = 1.66). Scatter plots of studentized residuals against unstandardized predicted values confirmed the homoscedasticity of residuals. All regression tolerances were greater than 0.1, indicating no multicollinearity. The P–P plot showed that the residuals approximately followed a normal distribution. The overall regression model was statistically significant (*F* = 6.19, *P* < 0.001, adjusted *R*^2^ = 0.17). BMI, EF, LVEDD, and sex were identified as significant independent variables influencing changes in GLS absolute values, with their effects being statistically significant (Table 4; Supplementary Fig. S2).

**Table 4:** Multivariable linear regression analysis of predictors of GLS in patients with PitNETs.

Variable	Regression coefficient	Standard error	95% confidence interval	*P*
BMI	−0.16	0.05	−0.25–0.06	0.00*
EF	0.11	0.04	0.03–0.19	0.00*
E/A	1.03	0.12	−0.26–2.33	0.12
E/e	0.20	0.10	−0.00–0.41	0.05*
LVDD	0.13	0.04	0.05–0.21	0.00*
Sex	1.01	0.40	0.21–1.80	0.01*
Handler	−0.41	0.43	−0.96–0.75	0.81
Interval	−0.01	0.37	−0.33–1.14	0.28

BMI, body mass index; EF, ejection fraction; LVEDD, left ventricular end-diastolic diameter.

* Statistical significance at *P* < 0.05.

### Subgroup analysis: acromegalic patients

To address cohort heterogeneity, an acromegaly-specific analysis (*n* = 164) was performed. The mean absolute GLS was −14.15% ± 2.80%. Multivariable linear regression, adjusting for sex and operator, showed that LVEDD (*β* = 0.12, *P* = 0.02) and IGF-1 levels (*β* = −0.001, *P* = 0.04) were independent predictors of impaired GLS, whereas BMI was not significant (*P* = 0.11). The overall model was statistically significant (adjusted *R*^2^ = 0.19, *F* = 4.9, *P* < 0.001), indicating that myocardial strain impairment in acromegaly is primarily driven by disease activity and LV remodeling rather than general metabolic burden (Table 5).

**Table 5:** Multivariable linear regression analysis in acromegaly subgroup (*n* = 164).

Variable	Regression coefficient (*β*)	Standard error	95% CI	*P*-value
BMI (kg/m^2^)	−0.10	0.06	−0.21–0.02	0.11
EF (%)	0.09	0.05	−0.01–0.19	0.07
LVEDD (mm)	0.12	0.05	0.02–0.22	0.02*
IGF-1 (ng/mL)	−0.001	0.0005	−0.002 to −0.00003	0.04*
Sex (F vs M)	0.85	0.48	−0.10–1.79	0.08
Operator (L vs C)	−0.35	0.50	−1.34–0.64	0.50

Model *F* = 4.9, *P* < 0.001; adjusted *R*^2^ = 0.19.

* Statistical significance at *P* < 0.05.

### Impact of surgical status on myocardial function

To evaluate potential bias from surgical outcomes, patients were stratified by surgical status and follow-up interval. Pre-operative patients (*n* = 58) had a mean GLS of −14.59% ± 2.85, whereas post-operative patients (*n* = 140) had a mean GLS of −14.13% ± 2.81. This difference was not statistically significant (*P* = 0.31).

Further analysis of the post-operative group based on follow-up interval revealed that patients evaluated >6 months after surgery (*n* = 79) had significantly worse GLS (−13.78% ± 2.77) compared with those assessed ≤6 months post-surgery (*n* = 61; −14.54% ± 2.85; *P* = 0.04), suggesting a delayed decline in myocardial function independent of acute surgical effects. These findings indicate that while immediate post-operative GLS remains similar to pre-operative values, long-term follow-up is necessary to detect subtle, time-dependent changes in myocardial strain (Table 6).

**Table 6:** Surgical status and follow-up impact on GLS.

Group	*n*	Mean GLS (%) ± SD	*P*-value (vs pre-op)
Pre-operative	58	−14.59 ± 2.85	-
Post-op ≤6 months	61	−14.54 ± 2.85	0.91
Post-op >6 months	79	−13.78 ± 2.77	0.04*

Note: significant late decline in GLS observed >6 months after surgery.

* Statistical significance at *P* < 0.05.

## Discussion

In this study, we utilized two-dimensional speckle-tracking echocardiography to identify subclinical left ventricular dysfunction in a cohort of patients with PitNETs. Our principal findings are threefold: first, GLS was significantly impaired in PitNET patients despite preserved ejection fraction; second, BMI, left ventricular dimensions, ejection fraction, and sex were independent predictors of GLS in the overall cohort; and third, a dedicated analysis of acromegalic patients revealed that disease activity (IGF-1 levels) was a significant predictor of worse myocardial strain, providing a disease-specific insight into cardiac impairment.

These cardiac findings must be interpreted within the specific context of our clinical cohort. The PitNETs, through hormone hypersecretion and mass effects, significantly disrupt pituitary function. Epidemiological studies indicate that non-functional PitNETs are the most common subtype (48.1%), followed by prolactinomas (27.8%) (17). However, in our surgical cohort, GH-secreting tumors were predominantly observed (90%), which reflects our hospital’s surgical focus. Acromegaly, caused by GH-producing PitNETs, is associated with high mortality and comorbidities, including cardiovascular risk (18). Our cohort’s mean age of 42 years aligns with the typical onset age for PitNETs, around 44 years (19). Female patients in our cohort were both more numerous and older on average (45.25 ± 13.63 years) than male patients (41.96 ± 12.53 years), with a greater proportion of female patients being diagnosed with functional PitNETs (20). Our finding that the most common age of diagnosis for both functional and non-functional PitNETs is between 30 and 50 years aligns with established epidemiological data, which consistently report a peak incidence in the 3rd to 5th decades of life (21). This pattern is further reflected in specific subtypes; for example, a large Chinese acromegaly cohort reported a mean age at diagnosis of ∼34.4 ± 11.7 years (22). These concordant findings support an emphasis on clinical vigilance for PitNETs in middle-aged adults.

We also identified four rare mixed-secreting PitNETs that presented with atypical symptoms such as hyperthyroidism and amenorrhea, which differed from the more common clinical features seen in GH-secreting PitNETs such as acromegaly, polyuria, and polydipsia. This highlights the diagnostic complexity of PitNETs, as their clinical presentations can overlap substantially across subtypes (23). Diagnoses in our cohort were confirmed primarily via endocrinology and neurosurgery referrals, with MRI serving as the primary detection method, particularly in cases with atypical presentations (Table 1) (24). These results underscore the need for comprehensive diagnostic tools to accurately differentiate between the various PitNET subtypes.

The relationship between disease activity and myocardial dysfunction in acromegaly appears complex. In our cohort, the relationship was complex; a model based on current GH and IGF-1 levels alone was not sufficient to predict GLS, whereas BMI was a significant predictor. This aligns with studies in treated acromegaly patients, where GH and IGF-1 were also not major determinants of residual cardiometabolic risk (25). This suggests that for detecting this specific cardiac complication, metabolic health status may provide more immediate insight than a single measurement of hormonal activity. The potential role of long-term hormone exposure, however, is highlighted by the trend we observed between a longer diagnostic interval and worse myocardial function.

In our study, 2D-STE revealed significantly reduced GLS values in functional and non-functional PitNET patients, which were lower than the usual lower limits for healthy controls (−6.9% for males, −18.5% for females) (16). However, there was no significant difference in GLS between the functional and non-functional PitNET groups. This suggests that GLS is a sensitive marker for myocardial dysfunction, but it may not distinguish between the different types of PitNETs. The lack of significant GLS difference between these subtypes could be attributed to shared underlying pathophysiological mechanisms, such as tumor mass effects and hormonal dysregulation, that led to similar cardiac alterations across PitNET types. This finding implies that GLS is more reflective of general myocardial dysfunction in PitNET patients rather than a specific marker of tumor functionality (26).

Significant correlations were found between GLS and LVEF, E/e' ratio, and BMI, reinforcing that these factors influence myocardial strain in PitNET patients. The positive correlation between GLS and LVEF indicates that GLS can complement traditional cardiac measures such as LVEF in detecting early myocardial dysfunction. The negative correlation between GLS and BMI suggests that higher BMI may exacerbate myocardial dysfunction, which aligns with previous studies indicating that obesity is a significant contributor to cardiovascular risk (27). Given the high prevalence of obesity in the PitNET cohort, particularly in functional PitNETs, our findings underscore the importance of weight management and metabolic health in improving cardiac function in these patients.

The multiple linear regression analysis identified BMI, EF, LV dimensions, and sex as independent predictors of GLS. Notably, BMI was the strongest predictor, which suggests that obesity plays a key role in determining myocardial strain in this cohort. The interval from disease onset to diagnosis was also included in the regression model, which may reflect the impact of prolonged hormone exposure and tumor mass effects on myocardial function. EF and LV dimensions also emerged as significant predictors, reinforcing their importance in cardiac assessment in PitNET patients. These results suggest that clinicians should consider BMI, cardiac parameters, and timing of diagnosis when interpreting GLS in PitNET patients (28).

One important finding in our study was the operator-dependent variability observed in GLS measurements. Operator C recorded higher GLS values than operator L, and a discrepancy was also noted in BMI, EF, and E/e’ values. This suggests that operator variability can affect the reliability and reproducibility of 2D-STE measurements. This variability highlights the need for standardized protocols and operator training to ensure consistent and accurate measurements, particularly when GLS is used for clinical decision-making (23). The operator-dependent differences found in our study underscore the importance of training and ensuring standardization across clinical settings to reduce measurement bias.

Although 2D-STE is a promising tool for detecting subclinical myocardial dysfunction in PitNET patients, there are limitations to consider. Postoperative pathological data were unavailable, which limited our ability to assess the influence of tumor volume and surgical outcomes on GLS. In addition, the single-center design and relatively small cohort restrict the generalizability of our findings. Future studies should incorporate larger, multi-center cohorts and include more comprehensive postoperative imaging and pathological assessments to validate these results (29, 30).

Our study clarifies the complex interplay between PitNETs and myocardial dysfunction through targeted subgroup analyses. However, when specifically analyzing patients with acromegaly, we found that disease activity (IGF-1 levels) and left ventricular remodeling (LV diameter) were independent predictors of impaired GLS, whereas BMI did not emerge as a significant predictor. This contrasts with the findings from the whole cohort, where BMI was a significant predictor, reflecting the broader metabolic burden across all tumor types. This finding underscores the necessity of evaluating cardiac function in the context of specific endocrine pathologies rather than considering PitNETs as a monolith (31, 32). Furthermore, our surgical stratification revealed that GLS was significantly worse in patients followed for more than 6 months post-operatively compared to those followed earlier. This suggests a potential for delayed myocardial adaptation or even late decline, indicating that the reversibility of acromegalic cardiomyopathy may be incomplete and that continued long-term echocardiographic surveillance is warranted.

This study has several limitations. First, its single-center, retrospective design may limit the generalizability of the findings to broader PitNET populations. Second, the absence of tumor volume measurements and detailed postoperative pathological data precluded assessment of their influence on myocardial function. Third, although 2D-STE is highly sensitive for detecting subclinical myocardial dysfunction, the technique relies on manual adjustments, introducing operator-dependent variability. Finally, the study’s design did not allow assessment of disease onset, surgical impact, or long-term postoperative outcomes. Future prospective, multi-center studies with more comprehensive tumor and clinical data are needed to validate and extend these findings.

Despite these limitations, this study demonstrates that 2D-STE is a sensitive tool for detecting subclinical left ventricular dysfunction in PitNET patients. We found that myocardial impairment is independently associated with BMI, left ventricular dimensions, ejection fraction, and sex. Crucially, our dedicated subgroup analysis revealed that in patients with acromegaly, disease activity (IGF-1 levels) is a significant predictor of worse myocardial strain, providing a powerful, disease-specific insight. Furthermore, the observed decline in GLS in patients followed long-term (>6 months) after surgery highlights the need for sustained cardiac surveillance. These findings confirm the clinical value of 2D-STE for early, personalized cardiac risk stratification in PitNET patients. Future longitudinal studies incorporating comprehensive tumor characteristics are warranted to define the role of GLS in guiding long-term cardiovascular management.

## Supplementary materials



## Declaration of interest

The authors declare that they have no known competing financial interests or personal relationships that could have appeared to influence the work reported in this paper.

## Funding

This work did not receive any specific grant from any funding agency in the public, commercial, or not-for-profit sector.

## Author contribution statement

Yinxia Li and Yifei Yu were responsible for methodology, formal analysis, investigation, and writing the original draft. Yanxin Xu was responsible for methodology, formal analysis, investigation, writing review and editing, and resources. Rui Shen was responsible for conceptualization, formal analysis, investigation, writing review and editing, funding acquisition, and supervision.

## Data availability

The datasets generated and/or analyzed during the current study are available within the manuscript and its supplementary materials.

## Ethics approval

This study was conducted in accordance with the Declaration of Helsinki and was approved by the Ethics Committee of Huashan Hospital, Fudan University (Approval No.: 2018002). Written informed consent was obtained from all participants.
