# Supplementary material for: Subclinical myocardial dysfunction in pituitary neuroendocrine tumor patients: a 2D speckle-tracking echocardiography study
Source: Endocr Connect. 2025 Dec 9;14(12):e250641. doi: 10.1530/EC-25-0641 (PMC12697240; doi:10.1530/EC-25-0641)
Supplement: Supplementary file 1 [file supplementary_materials.pdf]

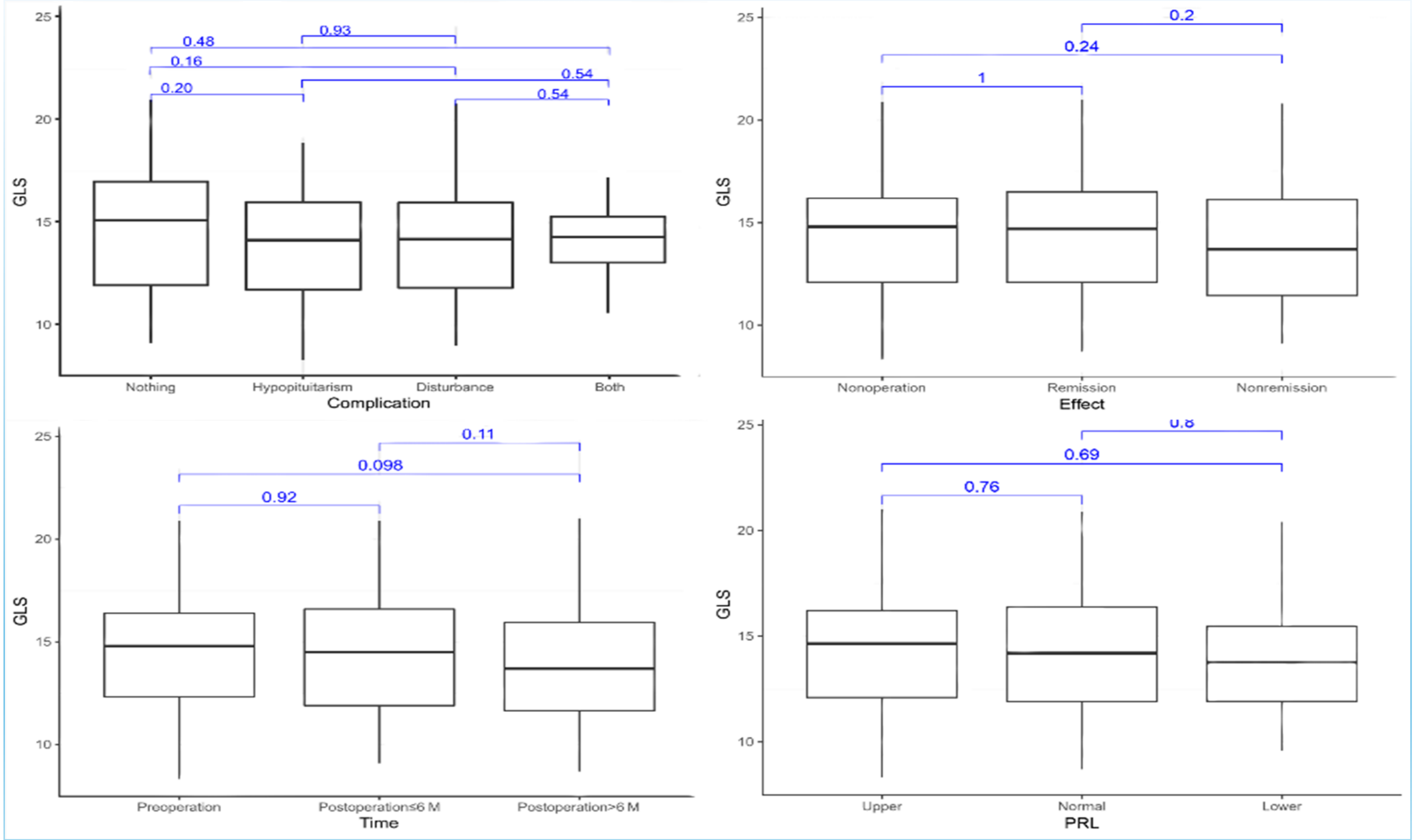

Supplementary data

Fig. S1 Plot of box comparison of mean GLS absolute values across multiple groups.

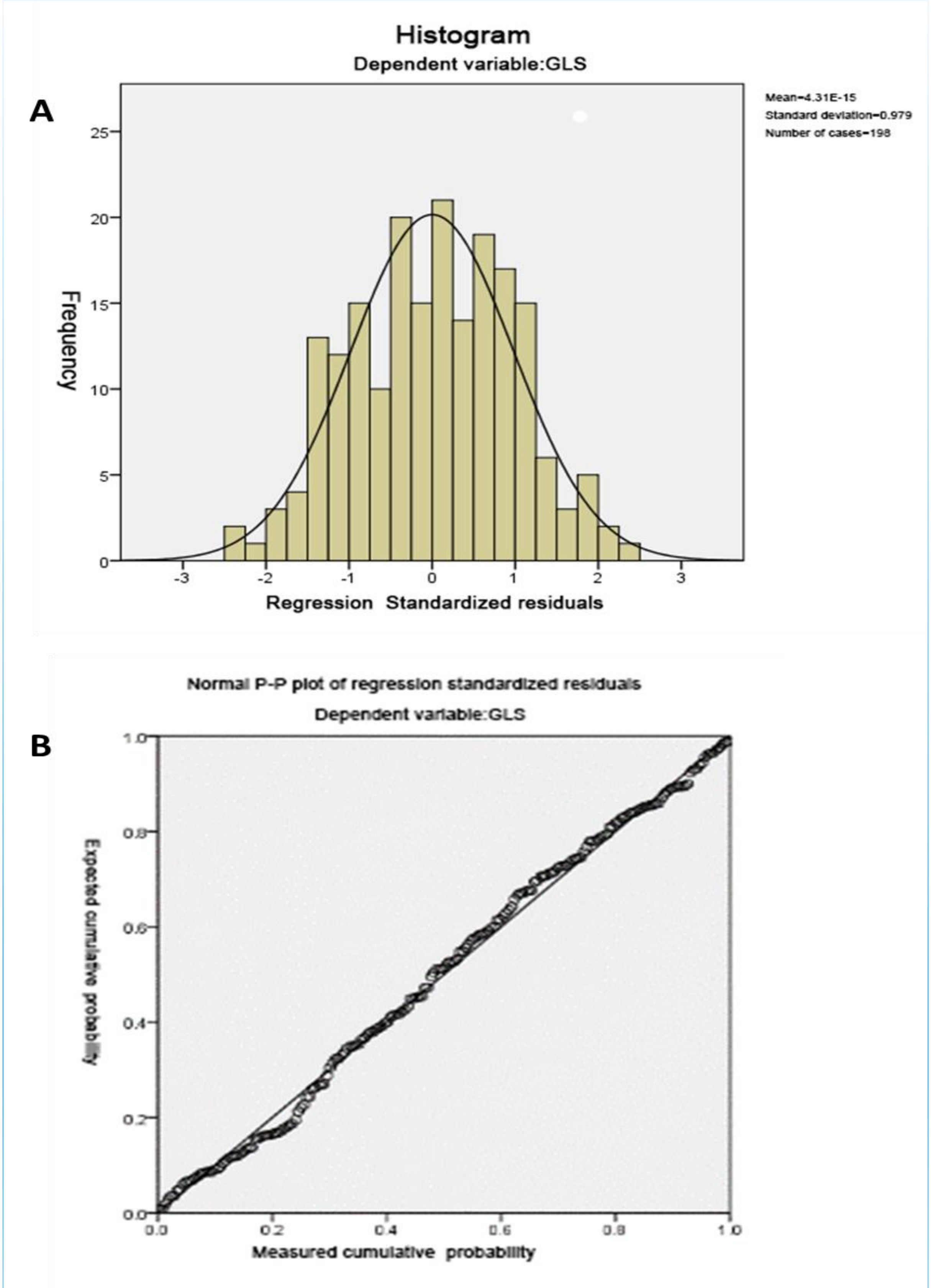

Fig. S2 Assessment of regression model assumptions: normality of residuals (histogram) and goodness-of-fit (P-P plot).
